# Homologous Expression of a Subcomplex of *Pyrococcus furiosus* Hydrogenase that Interacts with Pyruvate Ferredoxin Oxidoreductase

**DOI:** 10.1371/journal.pone.0026569

**Published:** 2011-10-24

**Authors:** R. Christopher Hopkins, Junsong Sun, Francis E. Jenney, Sanjeev K. Chandrayan, Patrick M. McTernan, Michael W. W. Adams

**Affiliations:** Department of Biochemistry and Molecular Biology, University of Georgia, Athens, Georgia, United States of America; Max-Planck-Institute for Terrestrial Microbiology, Germany

## Abstract

Hydrogen gas is an attractive alternative fuel as it is carbon neutral and has higher energy content per unit mass than fossil fuels. The biological enzyme responsible for utilizing molecular hydrogen is hydrogenase, a heteromeric metalloenzyme requiring a complex maturation process to assemble its O_2_-sensitive dinuclear-catalytic site containing nickel and iron atoms. To facilitate their utility in applied processes, it is essential that tools are available to engineer hydrogenases to tailor catalytic activity and electron carrier specificity, and decrease oxygen sensitivity using standard molecular biology techniques. As a model system we are using hydrogen-producing *Pyrococcus furiosus*, which grows optimally at 100°C. We have taken advantage of a recently developed genetic system that allows markerless chromosomal integrations via homologous recombination. We have combined a new gene marker system with a highly-expressed constitutive promoter to enable high-level homologous expression of an engineered form of the cytoplasmic NADP-dependent hydrogenase (SHI) of *P. furiosus.* In a step towards obtaining ‘minimal’ hydrogenases, we have successfully produced the heterodimeric form of SHI that contains only two of the four subunits found in the native heterotetrameric enzyme. The heterodimeric form is highly active (150 units mg^−1^ in H_2_ production using the artificial electron donor methyl viologen) and thermostable (t_1/2_ ∼0.5 hour at 90°C). Moreover, the heterodimer does not use NADPH and instead can directly utilize reductant supplied by pyruvate ferredoxin oxidoreductase from *P. furiosus*. The SHI heterodimer and POR therefore represent a two-enzyme system that oxidizes pyruvate and produces H_2_ *in vitro* without the need for an intermediate electron carrier.

## Introduction

The supply of cost-effective fossil fuels is finite, and for decades a major focus of research has been renewable energy generation [1]. Energy sources of the future must be abundant and carbon neutral with minimal impact on the environment. Driven by powerful new molecular biology tools, biofuel research has dramatically increased in the past decade, however, significant effort is still necessary to develop an economically viable, sustainable, and renewable energy supply [2], [3], [4]. As an energy carrier, hydrogen is attractive as it is non-toxic and has three times the energy of gasoline per unit mass [5]. Currently hydrogen is produced by steam reforming of natural gas or electrolysis of water, both of which are either non-renewable or inefficient on a large scale [5], [6], [7]. For sustainable and renewable production of hydrogen an abundant source of energy, such as sunlight, must be utilized. Photobiological production of hydrogen is an appealing solution but many problems remain in coupling oxygenic photosynthesis with the enzymatic production of hydrogen [3].

The ability to metabolize hydrogen is distributed across all three domains of life and is catalyzed by the hydrogenase enzymes [8]. Regardless of their source, these enzymes are usually highly regulated on the transcriptional level, require a complicated *in vivo* maturation process, and are inactivated by molecular oxygen. Two major classes of phylogenetically unrelated hydrogenases are known, nickel-iron (NiFe) and iron-iron (FeFe) [9], [10], and these catalyze the reversible interconversion of hydrogen, two protons and two electrons (Eqn. 1). These enzymes have been investigated for almost 80 years [11] but it has only recently become possible to manipulate or redesign the enzymes using standard molecular biology approaches [12], [13]. The FeFe enzymes have a limited distribution in the microbial world and although they typically have high catalytic rates of hydrogen production they are very sensitive to irreversible inactivation by molecular oxygen [9]. NiFe hydrogenases are ubiquitous in bacteria and archaea and function physiologically in both hydrogen oxidation and evolution [8]. They are much more resistant to molecular oxygen, and as such may be better targets for engineering, notwithstanding their lower catalytic turnover rates as compared to FeFe hydroenases (5–10%) [10], [14]. In order to link these enzymes to energetic biological processes, and exploit their ability to generate molecular hydrogen, it will be necessary to tailor catalytic activity, further reduce oxygen sensitivity, and even change coenzyme specificity.

2H^+^ + 2e^−^↔ H_2_ (1)

**Figure 1:**
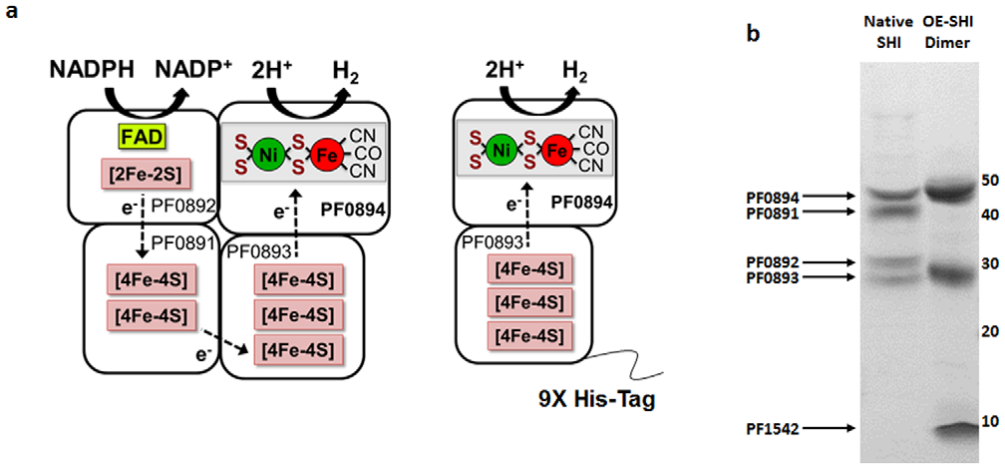
*Pyrococcus furiosus* heterotetrameric and dimeric soluble hydrogenase I. (**a**) Model of predicted cofactor contents of the heterotetrameric form of *Pf* SHI (taken without modification from Sun et al. [10]) and of the heterodimeric form of SHI that was produced (PF0893-PF0894) in this study that lacks PF0891 and PF0892. In the dimeric form, PF0893 is modified with an N-terminal His_9_ tag. The abbreviations used are: CO, carbonyl ligand; CN, cyanide ligand; FAD, flavin adenine dinucleotide; NADP, nicotinamide adenine dinucleotide phosphate. (**b**) SDS-PAGE of purified heterotetrameric (*left lane*) and heterodimeric form of SHI (*right lane*) with molecular masses indicated in kDa.

As a model organism we are investigating the hyperthermophilic archaeaon *Pyrococcus furiosus* (*Pf*), an obligate anaerobe that ferments simple and complex sugars to produce organic acids, CO_2_, and (in the absence of elemental sulfur) H_2_ [15]. *Pf* has three operons that encode NiFe hydrogenases; two cytoplasmic enzymes consisting of four subunits and a membrane bound hydrogenase (MBH) with 14 putative subunits [16], [17]. The two soluble enzymes, soluble hydrogenase I (SHI) and soluble hydrogenase II (SHII), utilize NAD(P)(H) as the physiological electron carrier [18], [19]. *Pf* SHI is a heterotetrameric enzyme consisting of the typical large (LSU PF0894) and small (SSU PF0893) subunits along with two additional subunits predicted to contain FeS clusters (PF0891) and a flavin in the form of FAD (PF0892) (Figure 1a). *Pf* SHI is a remarkably stable enzyme having a t_1/2_ at 90°C of approximately 12 hours and t_1/2_ after exposure to air of about 6 hours [18].

Recently, a genetic system was developed for *Pf* allowing the markerless disruption or integration of genes onto the chromosome [20]. This system marks a significant turning point in the ability to use *Pf* as a model organism. A host strain (COM1) was generated via the deletion of *pyrF* (orotidine 5′-monophosphate decarboxylase) which allows subsequent gene knockouts and marker excision. Using this strategy, knockout mutants of each of the two cytoplasmic hydrogenases, *shIβγδα* (soluble hydrogenase I), and *shIIβγδα* (soluble hydrogenase II) were constructed [20]. Expanding on this technique we utilize here a marked knock-in strategy to introduce an expression cassette into the *Pf* chromosome for homologous overexpression. To drive transcription of recombinant genes in *Pf* the promoter region of the gene encoding the S-layer protein (PF1399) was chosen. Based on microarray data ([21]) PF1399 is a high level, constitutively-expressed gene whose promoter will allow universal expression regardless of growth condition.

As a first step towards the production of ‘minimal’ hydrogenases, the goal of this work was to engineer a form of SHI that contained only two (LSU and SSU) rather than four subunits. This has been reported for the enzyme from some *Ralsonia* species but this was achieved by dissociation of the native tetrameric hydrogenase using electrophoresis and not by genetic manipulation [22]. In addition, we wished to take advantage of a new auxotrophic marker system for manipulating chromosomal DNA in the related organism *Thermococcus kodakarensis* [23], [24]. It was shown that the deletion of an essential gene *pdaD* (TK0149 arginine decarboxylase) could be complemented with addition of the polyamine precursor agmatine, a metabolite not found in complex growth media. Based on this work, we have devised a simple method of integrating genes of interest onto the chromosome of *Pf* using agmatine prototrophy as a marker and report herein on the expression and characterization of an active, stable heterodimeric subcomplex of *Pf* SHI.

## Results

**Figure 2:**
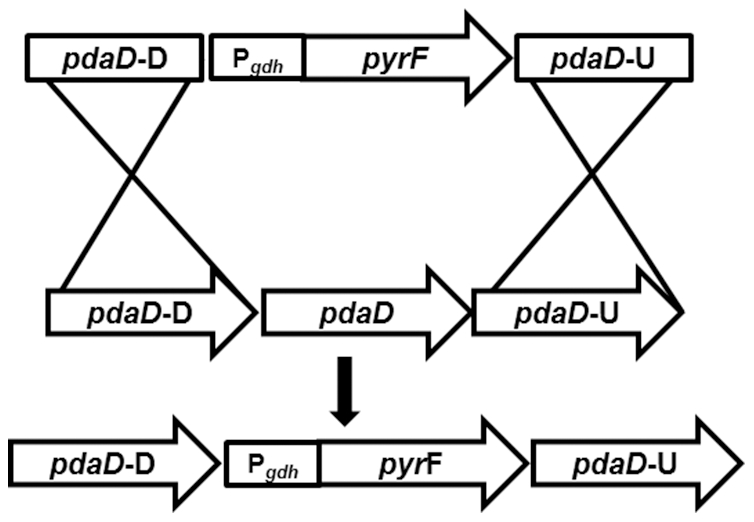
*pdaD* disruption with P*_gdh_ pyrF*. The 1kbupstream and downstream regions for the *pdaD* locus (PF1399) were cloned around the P*_gdh_ pyrF* cassette [20]. Using previously described transformation methods the *Pf* chromosome was disrupted at *pdaD* generating a uracil prototroph and agmatine auxotroph [20].

A *Pf* strain overexpressing the dimeric hydrogenase was constructed using an agmatine selection approach. It was recently reported that agmatine is essential for the growth of the hyperthermophilic archaeaon *Thermococcus kodakarensis* [23], a close relative of *Pf* that grows at a lower temperature (T_opt_ 85°C versus 100°C for *Pf*). Agmatine is derived from the decarboxylation of arginine and is a precursor for polyamine synthesis. In addition, it was recently discovered to be an essential conjugate of tRNA^Ile^ for AUA decoding in archaea [25]. Disruption of *Pf pdaD* (PF1623 arginine decarboxylase) with the P*_gdh_ pyrF* cassette (Figure 2) exhibited auxotrophy for agmatine and this allowed selection in the complex media used for *Pf* as yeast extract and casein both lack agmatine. This departure from defined media in growing and selection of *Pf* genetic mutants greatly simplifies the process of transformation.

**Figure 3:**
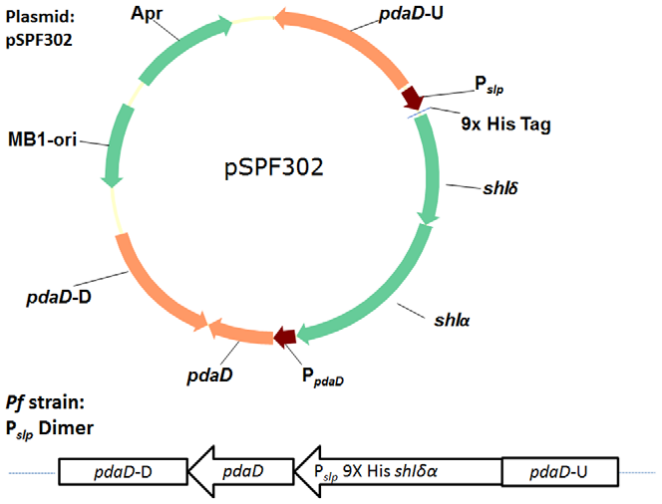
OE-SHI Dimer recombination plasmid and P*_slp_* Dimer strain. Plasmid pSPF302 was constructed for the integration of P*_slp_ shIδα* onto the *Pf* chromosome at the *pdaD* locus. After homologous recombination P*_slp_* Dimer strain contains the dimeric SHI construct under control of the constitutive promoter P*_slp_* and the *pdaD* with its native promoter.

A plasmid pSPF300 was designed to allow simple integrations on the *Pf* chromosome at the *pdaD* locus and includes a multiple cloning site after the P*_slp_* promoter for cloning of genes for homologous (or heterologous) expression in *Pf*. To investigate if *Pf* SHI can exist as a dimeric enzyme, the LSU and SSU (PF0893-0894) were cloned into the homologous recombination plasmid pSPF300 with the addition of an N-terminal His_9_-tag (on PF0893) generating plasmid pSPF302 (Figure 3). Linearized pSPF302 was successfully used to transform *Pf* and integrate the P*_slp_ shIδα* overexpression construct onto the *Pf* chromosome (Table 1).

**Table 1:** 

Strain	Genotype	Originating Strain	Source
DSM	Wild Type	DSM3638	15
ΔSHI	Δ*pyrF* Δ*shIβγδα*	COM1	20
ΔSHIΔ*pdaD*	Δ*shIβγδα* Δ*pdaD*::*pyrF*	ΔSHI	This study
P*_slp_* Dimer	Δ*shIβγδα* P*_slp_shIδα*	ΔSHIΔ*pdaD*	This study

*Pf* cells harboring the P*_slp_ shIδα* overexpression construct (*Pf* strain P*_slp_* Dimer) were used for purification. The overexpressed (OE-SHI) dimer was purified to homogeneity (Figure 1b) with a final specific activity of 106 U mg^−1^ (MV-linked hydrogen evolution) (Table 2), which is comparable to that obtained with the heterotetrameric enzyme [18]. Although only two subunits are expected from the purified enzyme complex, a persistent contaminant of approximately 8 kDa could not be separated from OE-SHI Dimer (Figure 1b). MALDI-TOF/TOF analysis revealed this to be PF1542, a gene annotated as snRNP (small nuclear ribonucleoprotein) that functions to mediate RNA-RNA interactions [26]. Size exclusion analysis of OE-SHI Dimer using a calibrated Superdex S200 column indicated the complex migrated at an apparent M_r_ of 88,000 daltons, in agreement with the trimer weight of PF0893-0894 and PF1542 (Table 3). As shown in Table 3, the OE-SHI Dimer was much less thermostable and more sensitive to oxygen exposure. As expected in the absence of the FAD-containing subunit PF0892, the OE-SHI Dimer was unable to evolve hydrogen from NADPH. Surprisingly, however, the OE-SHI Dimer was able to accept electrons from pyruvate via POR (Table 3). It has been previously reported that native *Pf* SHI cannot accept electrons from Fd_red_ using the POR-linked electron transfer system [27] and this was confirmed for our native SHI enzyme used in this study. Usually electrons derived from pyruvate are transferred from POR to the membrane bound hydrogenase via the cytoplasmic redox protein Fd [28]. In the *in vitro* assay OE-SHI Dimer was able to accept electrons directly from POR and the presence of Fd had no significant effect on activity. Native SHI is predicted to contain one [2Fe-2S] and five [4Fe-4S] clusters in addition to the NiFe active site (23 Fe total) while OE-SHI Dimer should only contain three 4Fe4S and the NiFe site (13 Fe total; Figure 1a). Accordingly, metal analysis showed that native SHI has a nickel to iron ratio of 1∶25 while the OE-SHI Dimer ratio is 1∶10 (Table 3).

**Table 2:** 

Step	Total Units (µmol min^−1^)	Total Protein (mg)	Specific Activity (U mg^−1^)	Yield (%)	Purification (-fold)
Cytoplasm	15300	5110	3	100	1
DEAE Sepharose	14660	2740	5	96	2
Nickel Sepharose 6	5560	53	106	36	35

**Table 3:** 

Property	Native SHI [12]	OE-SHI Dimer
Activity, MV-Linked (U mg^−1^)	163	145
Activity, NADPH-linked (U mg^−1^)	1	0
Activity, POR-linked (U mg^−1^)	0	0.2
Metal Content (Ni:Fe)	1∶25	1∶10
Apparent M_r_ (Daltons)	155,000	88,000
Stability at 90°C (t_1/2_, hr)	30	0.5
Stability in air (23°C, t_1/2_, hr)	25	4

## Discussion

The recent development of a genetic system in *Pf* [20] enables the deletion and homologous expression of genes, together with the tagging of proteins to facilitate purification. Moreover, the initial method using the *pyrF* deletion strain was limited by the use of defined media, as the standard complex media contain contaminating uracil (which overcomes the selection). The construction of the pSPF300 homologous recombination vector for integration at the *pdaD* locus provides a simple method for manipulating genes in *Pf* even in rich media. The pSPF300 vector includes the 1 kb regions for recombination, *pdaD* with native promoter (as marker), a high level, constitutive promoter (P*_slp_*) for the gene of interest, and a multiple cloning site containing four unique restriction sites. For routine overexpression of genes in *Pf* the agmatine auxotrophy based marker system and pSPF300 recombination vector provides a facile selection method.

Utilizing the *pdaD* marker system an N-terminal His_9_ dimeric version (PF0893-PF0894) of the heterotetrameric SHI (PF0891-PF0894) was cloned onto the chromosome of *Pf* to generate strain P*_slp_* Dimer (Figure 3) in a strain (ΔSHI) lacking the native enzyme. The ΔSHI deletion strain has already been characterized [20] and this was chosen as the parent strain since it might not be possible to introduce a dimeric SHI into *Pf* if the native SHI operon is still intact. Based on microarray data the promoter (P*_slp_*) for the gene (PF1399) encoding the highly expressed S-layer protein was used to drive transcription of a minimal form of the SHI enzyme. A phenotype was not observed for the P*_slp_* Dimer strain but this is not surprising as other hydrogenase deficient mutants of *Pf* also exhibited no obvious phenotype [20]. OE-SHI Dimer was able to accept electrons directly from POR *in vitro* and the possibility exist that the dimeric could short-circuit the path of electrons from POR to the membrane bound hydrogenase. Since this would bypass the creation of a proton motive force and the conservation of energy during metabolism one would expect a severe retardation of growth. P*_slp_* Dimer exhibited similar growth to wild-type *Pf* and it appears *in vivo* the flow of electrons remains unchanged.

OE-SHI Dimer was purified to near homogeneity utilizing a two-step ion-exchange and nickel sepharose 6 purification protocol (Figure 1b; Table 2). The persistent contamination of OE-SHI Dimer with PF1542 was unexpected as they do not share any similarity in predicted function. A subsequent size exclusion column was also unable to separate the proteins but the complex migrated at the expected M_r_ for a heterotrimer (Table 3). This suggests that OE-SHI Dimer and PF1542 form a stable complex but the reason for this association is not known. In combination with the N-terminal His tag and high constitutive expression, this protocol greatly simplifies purification and provides a superior yield of enzyme. For comparison, the original report of SHI purification [18] reported 11 mgs of pure enzyme obtained from 450 g *Pf* cells; OE-SHI Dimer was purified from 330 g cells for a final yield of 53 mgs, a more than ten-fold increase in yield.

**Figure 4:**
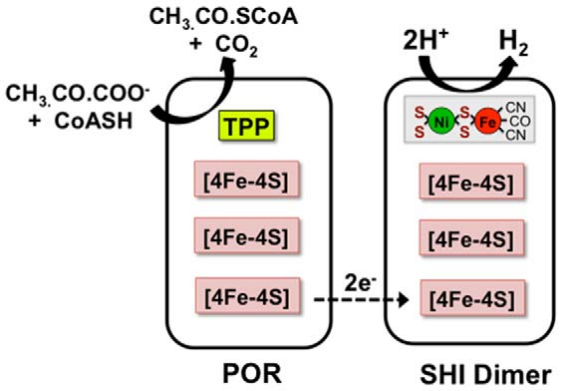
Model of the pyruvate-oxidizing, hydrogen producing POR-SHI Dimer system. The abbreviations are: POR, pyruvate ferredoxin oxidoreductase; SHI Dimer, heterodimeric form of SHI (PF0894+PF0893). TPP represents thiamine pyrophosphate.

The OE-SHI Dimer was markedly less stable than native SHI, but this is not unexpected as the absence of two partner subunits of the normally heterotetrameric enzyme would destabilize the complex. Although the heterodimeric enzyme appears less stable than native SHI after exposure to air and incubation at 90°C (under the same conditions of buffer and protein concentration, Table 3), the enzyme is still very robust as compared to mesophilic hydrogenases. The activity of the purified dimer with the redox dye MV was comparable to that of native SHI. As expected the OE-SHI Dimer lacked the ability to accept electrons from NADPH since the FAD-containing subunit PF0892 (and FeS-cluster containing subunit PF0891) are absent. Surprisingly, however, the OE-SHI Dimer was able to accept electrons directly from pyruvate ferredoxin oxidoreductase (POR), a reaction that native SHI cannot catalyze, and yet interestingly OE-SHI dimer cannot accept electrons from ferredoxin, the physiological electron acceptor of POR. Hence we have engineered a form of SHI that by chance directly interacts with native POR. This results in a two enzyme system that oxidizes pyruvate and produces H_2_ without the need for an intermediate electron carrier, such as ferredoxin or NAD(P). As shown in Figure 4, POR contains thiamine pyrophosphate and three [4Fe-4S] clusters and oxidizes pyruvate to acetyl CoA [29]. Presumably there is direct electron transfer between the iron-sulfur clusters of the two enzymes (Figure 4).

One of the goals in engineering hydrogenases is to change coenzyme specificity, and this was achieved in this case by simply deleting two subunits. Activities with physiological-relevant electron carriers such as NADPH are usually much less than that measured with the artificial electron donor MV, as is evident with native SHI (Table 3). Consequently, while the OE-SHI Dimer and the native SHI had comparable MV-linked hydrogen evolution activities, the OE-SHI Dimer exhibited only five-fold less activity with a physiological electron donor, in this case the enzyme POR, compared to native SHI and its true physiological partner, NADPH (Table 3). The expression of an active, dimeric form of SHI from *Pf* is a critical step towards engineering minimal hydrogenases. In conjunction with the genetic tools now available, the hydrogenase of *Pf* provides a robust model system for further engineering enzymes that will have utility in biohydrogen generating systems.

## Materials and Methods

Molecular biology techniques were performed as previously described [30]. *Pf* strains used in this study are listed in Table 1. *Pyrococcus furiosus* (DSM 3638) was cultured on liquid and solid support medium as previously described [20] with the addition of 4 mM agmatine (Sigma Chemical, St. Louis, MO) as necessary for genetic selections.

*Pf* strain ΔSHI [20] was used as the parent for this study. For markerless deletion of *pdaD* (PF1623, arginine decarboxylase) 1 kb DNA flanking regions upstream and downstream of PF1623 were cloned around the P*_gdh_ pyrF* cassette (Figure 2) obtained from plasmid pGLW021 [20] using overlapping PCR. Transformation and selection of knockout *Pf* strains were performed as previously described [20] to generate strain ΔSHIΔ*pdaD*.

For homologous overexpression of genes in *Pf* a promoter region (200 bp upstream PF1399) was selected based on microarray data to drive transcription. Across a wide range of conditions ([21]), PF1399 is constitutively expressed at a high level. Strains with disrupted *pdaD* were selected with defined medium lacking uracil and supplemented with 4 mM agmatine. For simple integrations on the *Pf* chromosome using agmatine prototrophy as a marker the plasmid pSPF300 was constructed. An *Nsp*I fragment was deleted from plasmid pSET152 removing the integrase gene to generate pSET-NS. From this pSPF101 was constructed by inserting the P*_gdh_ pyrF* cassette into *Pst*I/*Nhe*I digested pSET-NS, which removes the OriT region, to produce pSPF101. The 200 bp upstream region from PF1399 (P*_slp_*, promoter region for S Layer Protein) and a multiple cloning site were inserted into *Sac*II/*Sph*I digested pSPF101 to generate pSPF102. A 1.1 kb upstream region of PF1623 was cloned into *Sal*/*Nhe*I pSPF102 making pSPF107. A 1.83 kb fragment containing intact PF1623 operon (0.73 kb) and 1.1 kb of its downstream regionwas amplified by PCR and cloned into *Asc*I/*Sph*I digested pSPF107 generating pSPF300. To construct a homologous recombination vector for the expression of dimeric hydrogenase, a cassette with 9X His tagged PF0893-0894 fused behind P*_slp_* was first produced by overlap PCR, SacII/KpnI treated PCR product was then ligated with same enzymes treated pSPF300 to make pSPF302 (Figure 3). Transformation of *Pf* strain ΔSHI with AscI/PmeI linearized pSPF302 was performed and recombinant strains selected as previously described [20] to generate strain P*_slp_* Dimer (Figure 3).

Native *Pf* SHI enzyme was purified from wild type *P. furiosus* DSM3638 as previously described [16]. *Pf* strain P*_slp_* Dimer was grown in a 600L fermenter essentially as previously described [31] with the addition of 10 µM uracil. Harvested cells were flash frozen in liquid nitrogen and stored at −80°C. All purification steps were performed using strict anaerobic technique under an atmosphere of argon. Cell-free lysate was prepared from the P*_slp_* Dimer strain (330 g, wet weight) and DEAE (Diethylaminoethyl) anion exchange chromatography (GE Healthcare, Piscataway, NJ) performed as previously described [16]. Fractions eluting from DEAE anion exchange chromatography containing hydrogenase activity were pooled and loaded onto a 5 mL Ni Sepharose 6 Fast Flow column (GE Healthcare) equilibrated in 50 mM sodium phosphate, 300 mM sodium chloride, 2 mM dithiothreitol, pH 8.0 (Buffer A). A linear 20 column volume gradient of 0-500 mM imidazole in buffer A was applied to the column and resulting fractions were analyzed for hydrogenase activity (Table 2). Apparent M_r_ was measured using a calibrated Superdex S200 sizing column (GE Healthcare).

Hydrogenase activity was routinely determined by H_2_ evolution from methyl viologen (MV) (1 mM) reduced by sodium dithionite (10 mM) at 80°C as described previously [16], except the buffer was 100 mM EPPS, pH 8.4. One unit of hydrogenase specific activity is defined as 1 µmole of H_2_ evolved min^−1^ mg^−1^. For a physiologically relevant assay, methyl viologen and sodium dithionite were replaced by NADPH (1 mM) as described [16]. To investigate altered coenzyme specificity, physiological hydrogen evolution assays were performed as previously described [27]. Oxygen sensitivity assays were performed by exposing samples to air at 25°C. Thermal stability assays were measured by anaerobic incubation of the hydrogenase samples at 90°C. Residual enzyme activities were measured using the MV-linked H_2_-evolution assay. Metal content of enzyme samples was measured as previously described [12].
